# ^**18**^F-Fluorodeoxyglucose Positron Emission Tomography/CT Scanning in Diagnosing Vascular Prosthetic Graft Infection

**DOI:** 10.1155/2014/471971

**Published:** 2014-08-19

**Authors:** Ben R. Saleem, Robert A. Pol, Riemer H. J. A. Slart, Michel M. P. J. Reijnen, Clark J. Zeebregts

**Affiliations:** ^1^Division of Vascular Surgery, Department of Surgery, University Medical Center Groningen, University of Groningen, P.O. Box 30 001, 9700 RB Groningen, The Netherlands; ^2^Nuclear Medicine and Molecular Imaging, University Medical Center Groningen, University of Groningen, P.O. Box 30 001, 9700 RB Groningen, The Netherlands; ^3^Department of Surgery, Rijnstate Hospital, P.O. Box 9555, 6800 TA Arnhem, The Netherlands

## Abstract

Vascular prosthetic graft infection (VPGI) is a severe complication after vascular surgery. CT-scan is considered the diagnostic tool of choice in advanced VPGI. The incidence of a false-negative result using CT is relatively high, especially in the presence of low-grade infections. ^18^F-fluorodeoxyglucose positron emission tomography (^18^F-FDG PET) scanning has been suggested as an alternative for the diagnosis and assessment of infectious processes. Hybrid ^18^F-FDG PET/CT has established the role of ^18^F-FDG PET for the assessment of suspected VPGI, providing accurate anatomic localization of the site of infection. However, there are no clear guidelines for the interpretation of the uptake patterns of ^18^F-FDG as clinical tool for VPGI. Based on the available literature it is suggested that a linear, diffuse, and homogeneous uptake should not be regarded as an infection whereas focal or heterogeneous uptake with a projection over the vessel on CT is highly suggestive of infection. Nevertheless, ^18^F-FDG PET and ^18^F-FDG PET/CT can play an important role in the detection of VPGI and monitoring response to treatment. However an accurate uptake and pattern recognition is warranted and cut-off uptake values and patterns need to be standardized before considering the technique to be the new standard.

## 1. Introduction

Vascular prosthetic graft infection (VPGI) is an uncommon complication after reconstructive vascular surgery with an incidence ranging between 1 and 6%. The incidence of VPGI varies according to the bypass localization with less than 1% in case of subrenal aortic bypass, 1-2% after aortofemoral bypass, and up to 6% in case of infrainguinal bypass [1–3]. Nevertheless, it is one of the most difficult challenges faced by the vascular surgeon and is associated with devastating complication such as limb amputation (5–25%) and mortality rates as high as 25–88% [1–3]. The first and principal dilemma in clinically suspected VPGI is to obtain definite proof of the graft infection. Positive cultures either from percutaneous aspirated perigraft fluid or from surgically obtained material are considered by many the gold standard for VPGI but in clinical practice are often difficult to obtain. For adequate treatment, it is important to diagnose graft infection at an early stage. Unfortunately, clinical signs are variable and often subtle. They may include recurrent fevers and chills, back or groin pain, erythema, swelling, or a pulsatile mass in the groin, thus making the correct diagnosis sometimes tedious [4]. The commonly used first methods to evaluate and diagnose a VPGI are evaluation of elevated infection parameters in peripheral blood samples (erythrocyte sedimentation rate, white blood cell count, and C-reactive protein (CRP)), duplex ultrasound scanning, computed tomography (CT) scanning, and magnetic resonance imaging (MRI). However, the predictive value for diagnosing VPGI with either one of these diagnostic tools has proven to be relatively low [5]. To date CT is considered the gold standard in diagnosing VPGI because of its high spatial resolution providing a detailed view of the vascular structures and perivascular spaces. Diagnostic signs for VPGI on CT include the presence of local fluid, perigraft retention, pseudoaneurysm formation, and focal bowel thickening and air bubbles [6], though these findings are present in just 50% of VPGI cases and are even considered normal findings in the early postoperative period. Other CT findings suggesting a VPGI include thickening of the graft wall, adjacent blurred fat, and soft tissue swelling. CT scan sensitivity and specificity are claimed to be 95% but this high percentage can only be reached in clinically high suspected VPGI [7–9]. CT scan is much less reliable in case of a low-grade infection, with a sensitivity and specificity of 55% and 100%, respectively [10–12]. In particular, the false-positive results may lead to unnecessary surgery or lengthy antibiotic use while the false-negative results may have life or limb threatening consequences. Since 1996, several studies have evaluated the usefulness of 18-F-Fluoro-D-deoxyglucose positron emission tomography (^18^F-FDG PET) in the detection of infectious foci and, more specific, the usefulness in the analyses of suspected VPGI (Table 1). This review aims to summarize the accuracy and interpretation of ^18^F-FDG PET in the detection of VPGI.

## 2. Search 

A literature search was carried out using the following databases: Pubmed, the Cochrane library, Science Direct, and Embase. Two authors (Ben R. Saleem and Robert A. Pol), independently of each other, identified the studies for inclusion based on title or abstract using the following medical subject headings (MeSH): “positron emission tomography,” “blood vessel prosthesis,” and “infection.” These MeSH terms were applied in various combinations using the Booleans operators AND or OR. Papers were included from the start of the databases until January 2014. Reference lists from the selected articles were manually checked for additional relevant studies. In Pubmed, the “related article” algorithm was employed to identify additional articles. Case reports and short case series (number of patients < 4) were excluded from further review. In total, six original studies were selected and listed in Table 1.

## 3. ^18^F-FDG PET Imaging in Infectious Diseases 

**Figure 1:**
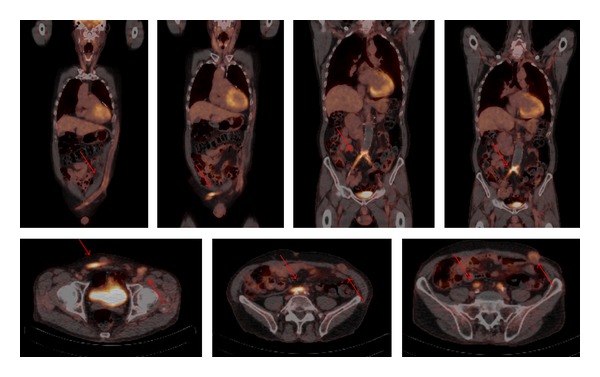
Coronal and axial view of fused ^18^F-FDG PET/CT images. In this particular case, a 67-year-old male patient underwent an aorto-bi-iliac bypass 16 years ago. After 3 years the bypass was revised because of an occlusion. Five years later the second bypass also occluded and an axillobifemoral bypass was constructed. Unfortunately this bypass also occluded twice. Patient is admitted to the hospital because of pain and redness at the level of the axillobifemoral bypass probably due to infection. ^18^F-FDG PET/CT scanning showed increased FDG uptake at the level of both occluded bypasses, the aorto-bi-iliac bypass and the axillobifemoral bypass. Arrows are pointing at increased FDG uptake in both bypasses. Ultimately, no bacteria were cultured.

^18^F-FDG PET imaging is based on uptake of radioactive-labeled glucose (^18^F-FDG) in metabolically active cells. Activated inflammatory cells, like malignant cells, predominantly metabolize glucose as a source of energy. In the stimulated state, inflammatory cells, such as neutrophils and macrophages, express high concentrations of glucose transporters that facilitate the movement of FDG through the cell membrane. The initial application was designed to diagnose cancer as increased glucose metabolism is often present in tumor cells, resulting in a higher ^18^F-FDG uptake than in the surrounding tissues [18]. However, it appeared that inflammatory and infectious lesions sometimes caused false-positive results. In the early years of clinical ^18^F-FDG PET imaging in oncology, cases of false-positive uptake in a wide variety of infections were described [19]. Although initially interpreted as false-positive results and even a disadvantage of the technique, its usefulness in detecting inflammation was further explored with respect to the potential of ^18^F-FDG PET imaging in different types of infection and inflammation [20, 21] (Figure 1). To increase the sensitivity and specificity FDG-PET can be fused with CT and or MR images. Hybrid PET/CT has been increasingly applied during the last decade [22]. ^18^F-FDG PET/CT can identify infections and their relation to the surrounding anatomy. Hybrid PET/MR has rapidly increased in interest in daily clinical practice. PET/MR also provides the anatomic information but with much higher soft tissue contrast and without the additional radiation dose from CT [23, 24]. To date the majority of reports on the role of ^18^F-FDG PET and fused ^18^F-FDG PET/CT imaging in the diagnosis of VPGI are case series and case reports of single patients [10, 13–17, 25, 26].

## 4. Accuracy of ^18^F-FDG PET in VPGI

The sensitivity and specificity of ^18^F-FDG PET vary greatly in current literature. Table 1 summarizes data on the use of ^18^F-FDG PET and fused ^18^F-FDG PET/CT imaging in suspected VPGI. In a large case series of 33 patients that compared the feasibility of ^18^F-FDG PET with CT, the sensitivity and specificity of ^18^F-FDG PET were, respectively, 91 and 64% [10]. Comparably, if no better results were published by Keidar et al. they prospectively assessed 39 patients (69 vascular prosthetic grafts) with suspected VPGI by ^18^F-FDG PET fused with CT. ^18^F-FDG PET/CT results were true positive in 14, false negative in 1, and false positive in 2 cases, resulting in sensitivity and specificity rates of 93% and 91%, respectively [13]. Spacek et al. also evaluated fused ^18^F-FDG PET/CT images in 76 patients (96 vascular prosthetic grafts) with suspected VPGI [15]. Although in this paper the specificity was similarly high with 93%, the sensitivity of 78% was remarkably low. The most recent study by our group evaluated both ^18^F-FDG PET and fused ^18^F-FDG PET/CT imaging. In a retrospective study a sensitivity of 93% and a specificity of 70% of ^18^F-FDG PET in 25 patients with clinically suspected VPGI were found [16]. We found no differences regarding sensitivity or specificity when fused ^18^F-FDG PET/CT images were assessed for their accuracy in diagnosing VPGI. Also no differences have been found in the literature yet on accuracy of ^18^F-FDG PET/CT between abdominal versus thoracic aorta graft infection [17].

## 5. Interpretation and Grading of the ^18^F-FDG PET Findings

There is no consensus with respect to the interpretation of the ^18^F-PET findings in the reviewed studies wherein a visual grading scale was used. Different variants such as a four- or five-point scale were used [10, 16]. The five-point scale was structured as follows: grade 0, FDG uptake similar to that in the background, grade 1, with low FDG uptake, comparable with that by inactive muscles and fat, grade 2, with moderate FDG uptake, clearly visible and higher than the uptake by inactive muscles and fat, grade 3, with strong FDG uptake, but distinctly less than the physiologic uptake by the bladder, and grade 4, with very strong FDG uptake, comparable with the physiologic urinary uptake by the bladder. Using this classification, lesions with grades 3 and 4 uptake are considered infected lesions [20]. The four-point grading scale consists of grade 1, with FDG uptake similar to that in the background, grade 2, with low FDG uptake, comparable with that by inactive muscles and fat, grade 3, with moderate FDG uptake, clearly visible and higher than the uptake by inactive muscles and fat, but distinctly less than the physiologic uptake by the bladder, and grade 4, with strong FDG uptake, comparable with the physiologic urinary uptake by the bladder. Lesions with grades 3 and 4 uptake were classified as high probability of VPGI. Besides these four- and five-point scales, other interpretations of FDG uptake have also been used. Keidar et al. defined an infectious process if focal increased ^18^F-FDG uptake in the region of any of the vascular grafts had a higher intensity than the surrounding tissues [13]. Spacek et al. interpreted FDG uptake as intense, inhomogeneous, or no uptake [15]. They considered intense focal FDG uptake as a positive result for VPGI. However, when inhomogeneous uptake was also considered as a positive result, then the sensitivity increased but the specificity decreased. The subgroup of patients with inhomogeneous FDG uptake represents the big challenge, because of nondiagnostic results. Spacek et al. suggested that a mild inhomogeneous FDG uptake might be explained either by infection of very low grade in which only a weak immune reaction might be anticipated or in immunocompromised patients [15]. Hybrid ^18^F-FDG PET/CT was found as a promising diagnostic tool in patients with inhomogeneous FDG uptake with the morphological appearance of the graft boundary [15]. Because a physiologic uptake is often visible in or around vascular prostheses, patterns of interpretation have been discussed. It is believed that a linear, diffuse, and homogeneous uptake is not likely to represent infection whereas focal or heterogeneous uptake with projection over the vessel on CT is highly suggestive of infection [10]. ^18^F-FDG PET scans can also be assessed using (semi) qualitative parameters (maximal standardized uptake value (SUVmax) and tissue-to-background ratio (TBR)). The TBR ratio can be calculated by dividing the SUVmax in the graft material by the mean blood-pool activity [16]. More recently, a semiquantitative method has been suggested for the evaluation of thoracic prosthetic graft infections in which a standard uptake value-max >8 in the perigraft area was considered the cut-off value for distinguishing between an infected graft and a noninfected graft [17]. Until now there are no clear guidelines for the interpretation of the uptake patterns of ^18^F-FDG as a signal for VPGI. Apparently the interpretation of the images by a visual grading scale is predominantly subjective. Besides visual and semiquantitative analyses, a quantitative analysis can be performed in which it is possible to calculate the curve of the arterial FDG concentration plotted against time (arterial input function). In this way physiological parameters can be measured in absolute units (e.g., glucose metabolic rate in mol min^−1^ g^−1^ or blood flow in ml min^−1^ g^−1^). Such absolute quantification is usually not performed in the clinical routine. It requires direct sampling of arterial blood with serial measurements and dynamic acquisition. Some noninvasive alternatives have been studied; the input function can be retrieved from the PET images using the aorta or using volumes of interest and partial volume correction.

## 6. False-Positive Interpretation of Images and Graft Physiological Uptake Patterns

**Figure 2:**
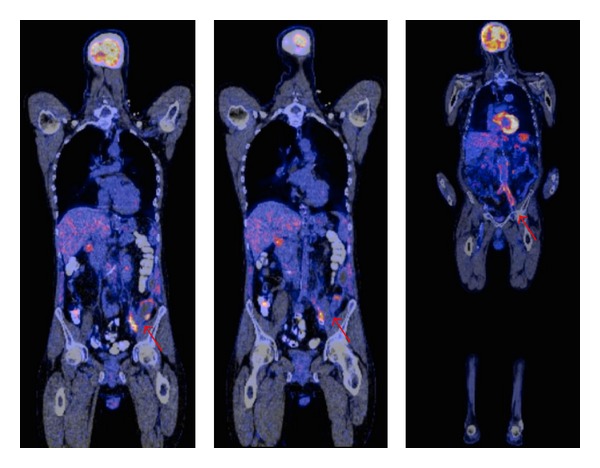
Coronal view of fused ^18^F-FDG PET/CT image of a culture proven not infected vascular prosthetic graft. In this particular case, a 45-year-old male patient underwent emergency surgery by placement of a Viabahn in the left common iliac artery because of rupture. He was admitted to the hospital 6 weeks later with fever and sepsis. ^18^F-FDG PET/CT scanning showed increased FDG uptake at the level of the left iliac artery. Ultimately, no bacteria were cultured from the graft under antibiotic therapy.

Increased ^18^F-FDG uptake may occur in postsurgical inflammatory changes, scar tissue, and native vessels [3, 13, 27]. The chronic aseptic inflammation due to the synthetic graft material, mediated primarily by macrophages, fibroblasts, and foreign-body giant cells, constitutes a potential base for ^18^F-FDG uptake even a long time after surgery, up to 16 years, depending on the used prosthetic material [28–30]. Within the first 6 to 8 weeks after surgery a physiological ^18^F-FDG graft uptake can result in a false-positive scan [2, 15, 17] (Figure 2). These physiological uptake patterns of ^18^F-FDG in synthetic vascular graft have been reported in both symptomatic and asymptomatic patients and render a diagnosis of an early VPGI extremely difficult. In a recent study patients who underwent an ^18^F-FDG PET/CT scan for other reasons than suspected graft infection were analyzed and a 3-grade arbitrary scale was used, high, low, or no uptake, to indicate ^18^F-FDG accumulation within the graft. Five circular regions of interest were drawn at different levels of the descending and suprarenal abdominal aorta to measure the SUVmean. The TBR was calculated by dividing the maximum SUV in the graft material by the mean blood-pool activity. Elevated ^18^F-FDG was seen in 10 out of 12 patients with synthetic abdominal aortic grafts after open reconstruction and in 1 out of 4 grafts after endovascular repair. Only 1 patient eventually developed a VPGI and thus the authors concluded that the risk of a false-positive diagnosis of VPGI by ^18^F-FDG PET/CT shortly after surgery is high [31]. More recently, a retrospective evaluation of the incidence and patterns of ^18^F-FDG uptake in 107 noninfected vascular grafts was published [32]. A 12-year ^18^F-FDG PET/CT scan database was searched for cancer patients with a history of vascular surgery with a synthetic graft. Again the SUVmean was measured in each graft and the pattern of uptake for each graft was recorded as focal, diffuse homogeneous, inhomogeneous, or absent. Dacron grafts had a significant higher metabolic activity than Gore-Tex grafts and native vein grafts. Also grafts used for a central reconstruction had a higher ^18^F-FDG uptake compared to grafts anastomosed in the groin or lower limb, most likely because Dacron is most commonly used during central reconstruction. Furthermore, the authors found a diffuse ^18^F-FDG uptake in 92% of noninfected vascular prostheses. The intensity of ^18^F-FDG uptake however was independent of the period after surgery, ranging from 5 months to 16 years with an average of 10 years [32].

## 7. ^18^F-FDG Uptake Patterns in Diabetes Mellitus Patients

Diabetes mellitus (DM) or elevated serum glucose levels and its influence on the sensitivity, specificity, and the accuracy of ^18^F-FDG studies are a controversial issue. Glucose metabolism can be disturbed in DM patients and therefore result in an increased uptake of ^18^F-FDG. A number of animal and human studies have shown that plasma glucose competes with ^18^F-FDG uptake in tumors. It therefore has been postulated that hyperglycemia may reduce and impair ^18^F-FDG uptake in malignant lesions [33–35]. Only a few studies are available on the role of DM in ^18^F-FDG uptake in infectious or inflammatory diseases. A recent study concluded that the incidence of false-negative scans in patients assessed for suspicion of an infectious or inflammatory process was not adversely affected between patients with or without DM and with high or normal serum glucose levels at the time of scanning [36]. While not affecting the accuracy when assessing infection, hyperglycemia during ^18^F-FDG PET scan in the oncology group may lead to higher false-negative rate and should be therefore avoided [36, 37]. For further interpretation, it is pertinent that FDG-PET reflects glucose metabolism not only in pathological conditions but also in various physiological conditions which can cause an increase in ^18^F-FDG. These issues require further research in order to improve the interpretations of ^18^F-FDG PET in diagnosing VPGI possible.

## 8. Uptake Patterns and Causative Organisms

*Staphylococcus* species are the most common causative organisms of aortic graft infection, mostly due to bacterial contamination at the time of graft placement.* Staphylococcus epidermidis* is a slowly growing, slime-producing organism classically causing a late, indolent VPGI [1, 38, 39]. It is estimated that* Staphylococcus aureus* species account for approximately 25% to 50% of al VPGI [40]. No studies are available about the differences in uptake patterns of ^18^F-FDG in VPGI caused by different species. The ^18^F-FDG PET characteristics of* Staphylococcus aureus* osteomyelitis and foreign-body-associated* Staphylococcus epidermidis* infections have been studied in rabbits [41].* Staphylococcus epidermidis* was found to reflect a low virulence of the pathogen and limited leukocyte infiltration, which was characterized by low ^18^F-FDG uptake.

## 9. Monitoring Response to Treatment with ^18^F-FDG PET/CT

Recently, the combined guidelines from the European Association of Nuclear Medicine (EANM) and the Society of Nuclear Medicine and Molecular Imaging (SNMMI) were published for the use of ^18^F-FDG PET in inflammation and infection [42, 43]. Based on cumulated reported accuracies, more than 85% of these guidelines state that the major indications for the use of ^18^F-FDG PET/CT in infection and inflammation are sarcoidosis, peripheral bone osteomyelitis, spondylodiscitis, evaluation of fever of unknown origin, and the primary evaluation of vasculitis [44]. For VPGI, it remains unclear if ^18^F-FDG PET/CT offers advantages over other imaging techniques yet, based on the available published data [40–44]. However, ^18^F-FDG PET/CT can not only be used to diagnose VPGI but it could also be used as a monitor of treatment response. ^18^F-FDG PET/CT seems to be useful for the diagnosis and also for therapy evaluation in vasculitis, sarcoidosis, and spondylodiscitis [44]. Other well-described applications, but without sufficient evidence-based indication, included suspected infection of intravascular devices, pacemakers, and catheters [45–48]. However to date there are no studies which show the benefits of ^18^F-FDG PET/CT as a monitoring tool for VPGI.

## 10. Discussion

**Figure 3:**
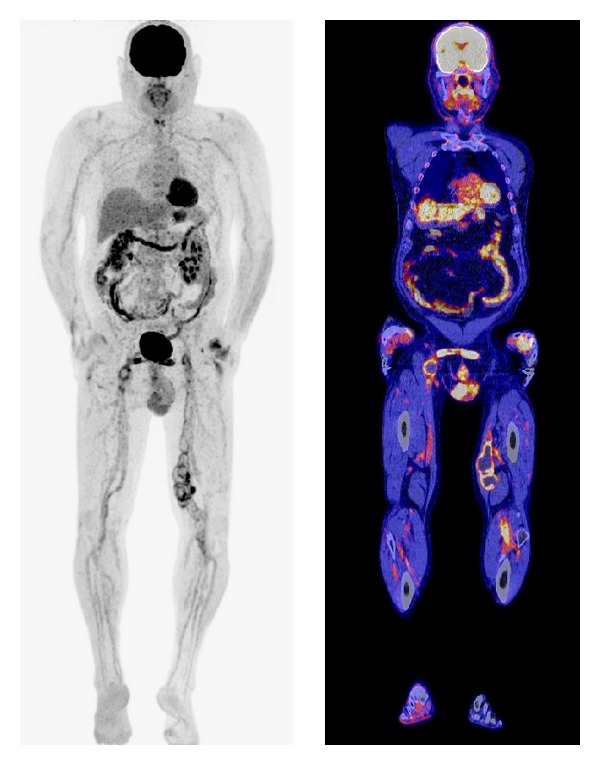
Coronal view of a fused ^18^F-FDG PET/CT image of a culture proven infected vascular prosthetic graft. In this particular case, a 70-year-old male patient underwent an ileofemoral bypass from the left to right side using Dacron. After 10 years the patient was readmitted with an occlusion of the bypass possibly due to a suture aneurysm. CT scan revealed an occlusion of the bypass with no signs of infection. ^18^F-FDG PET/CT scanning clearly showed increased FDG uptake at the level of the suture aneurysm. Ultimately,* Escherichia coli* were cultured from the graft.

**Figure 4:**
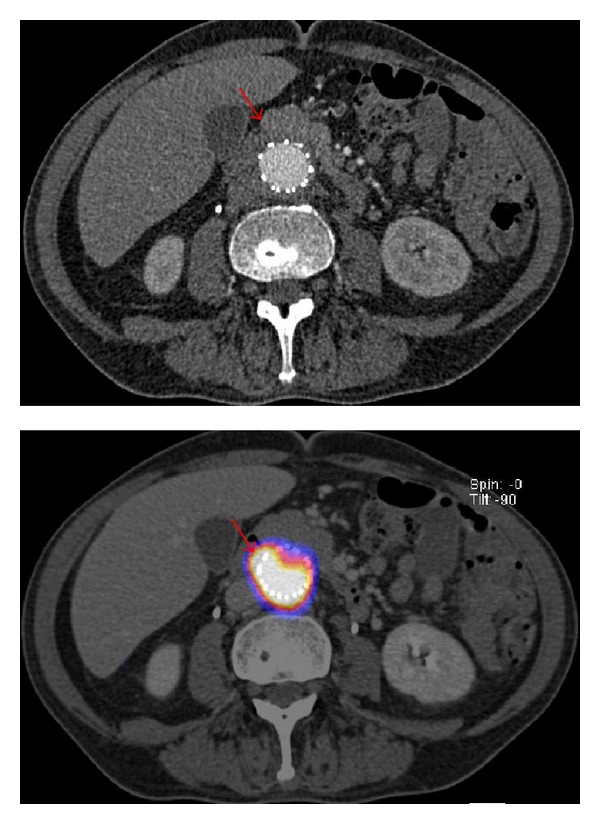
Axial view of a CT scan and fused ^18^F FDG PET/CT image of a culture proven infected vascular prosthetic graft. In this particular case, a 67-year-old male patient underwent an urgent endovascular procedure of a contained ruptured abdominal aortic aneurysm using a Cook Zenith prosthesis. After 7 months the patient was readmitted with clinical signs of a prosthetic infection. CT scan was assessed as a possible prosthesis infection. An ^18^F-FDG PET/CT scan confirmed the diagnosis by showing an increased FDG uptake at the level of the endograft. Ultimately,* Escherichia coli* were cultured from the graft.

This review has shown that ^18^F-FDG PET and ^18^F-FDG PET/CT may play an important role in the detection of VPGI. However, to date, there is only limited information regarding the accuracy of ^18^F-FDG PET/CT imaging in the detection of these infections. There are no clear guidelines for the interpretation of ^18^F-FDG PET images made for suspicion of VPGI. Because physiologic uptake is often visible in/around vascular prostheses, the specific patterns of uptake appear to be crucial. Based on the available literature it is suggested that a linear, diffuse, and homogeneous uptake should not be regarded as an infection whereas focal or heterogeneous uptake with a projection over the vessel on CT is highly suggestive of infection (Figures 3 and 4). However, low-grade infection may be presented as mild inhomogeneous of FDG uptake [15]. In case of inhomogeneous FDG uptake, we suggest a follow-up policy. ^18^F-FDG PET scan can not only be fused with CT scan. Recent studies show the benefits of fusing PET with MR scan. Possible additional advantages of PET/MR over PET/CT are the potential for motion correction, as well as reconstruction driven by anatomic information. The combination of PET radiopharmaceuticals and PET/MR imaging could significantly improve the sensitivity and specificity of the diagnosis and follow-up treatment of infectious and inflammatory diseases. It would allow for more accurate assessment of the extent and exact localization of inflammatory lesions than PET alone or PET/CT, especially in soft tissues that are prone to movement artifacts, for example, in vascular and cardiac infections and inflammatory bowel disease [24]. The development of PET/MR has triggered a shift in the PET detector technology and image correction paradigms. Most of the technical challenges have been solved, but clinical studies are required to show the areas of patient care for which PET/MR has advantages over other diagnostic methods.

With this current knowledge we recommend to further develop semiquantitative and quantitative analysis methods for the assessment of the FDG-PET images. One of the semiquantitative values is SUV. It is suggested in the literature that SUVmax >8 in the perigraft area appears to be the cut-off value for distinguishing infected grafts from noninfected grafts. However, this was based on a small number of patients and probably the SUV measurement was not based on the ENAM and SNMMI guidelines which makes it difficultly reproducible in other centres. ^18^F-FDG PET is generally assessed using visual criteria, looking for a focally increased uptake that may be compatible with infection. It is unclear if semiquantitative measurements such as SUV will contribute to the assessment, partly because of the considerable variability in the methodology used. This recommendation is an attempt to increase uniformity of ^18^F-FDG PET investigations in multicentre studies and for routine clinical applications [43]. It is therefore also essential that the used equipment is comparable. Besides the development of a quantitative analysis and to minimize the false-negative and false-positive interpretations, an experienced department of nuclear medicine is needed to analyze the ^18^F-FDG PET/CT scan. Hyperglycemia and DM have been previously considered as one of the main reasons for false-negative ^18^F-FDG PET/CT studies. However, based on the available literature, this appears to be an overestimated problem in which the false-negative rate in patients assessed for VPGI is not statistically significantly different between patients with and without hyperglycemia.

In conclusion,^ 18^F-FDG PET and ^18^F-FDG PET/CT can play an important role in the detection of VPGI and monitoring response to treatment; however, an accurate uptake and pattern recognition is warranted. Standardization of techniques and definitions of the cut-off uptake values and patterns are needed before recommending the technique as the new gold standard.

## Figures and Tables

**Table 1:** Summary of literature data regarding the use of ^18^F-FDG PET imaging requested in suspected vascular graft infection.

Study	Year	Study Design	Number of patient's	Imaging modality	Interpretation criteria	TP^1^	TN^2^	FP^3^	FN^4^	Sens∗ %	Spec∗∗ %
Fukuchi et al. [10]	2005	prospective	33	PET	Semiquantitative^a^	10	14	8	1	91	64
Keidar et al. [13]	2007	prospective	39	PET/CT	Visual	14	22	2	1	93	91
Lauwers et al. [14]	2008	case series	4	PET	Visual	3	0	1	0	—	—
Spacek et al. [15]	2009	prospective	76	PET/CT	Semiquantitative^b^	54	31	10	1	78.2	92.7
Bruggink et al. [16]	2010	retrospective	25	PET and PET/CT	Semiquantitative^c^	15	10	0	0	93^†^	70^†^
Tokuda et al. [17]	2013	retrospective	9	PET/CT	Semiquantitative^d^	4	5	0	0	—	—

^1^True positive.

^2^True negative.

^3^False positive.

^4^False negative.

∗Sensitivity.

∗∗Specificity.

^a^Five-point scale intensity of FDG uptake.

^b^Three point scale intensity of FDG uptake.

^c^Four point scale intensity of FDG uptake.

^d^SUVmax cut off value.

^†^ Results for both FDG-PET and fused FDG-PET-CT, judged by a nuclear medicine physician.

## References

[1] Saleem BR, Meerwaldt R, Tielliu IFJ, Verhoeven ELG, van den Dungen JJAM, Zeebregts CJ (2010). Conservative treatment of vascular prosthetic graft infection is associated with high mortality. *The American Journal of Surgery*.

[2] Legout L, D'Elia PV, Sarraz-Bournet B (2012). Diagnosis and management of prosthetic vascular graft infections. *Medecine et Maladies Infectieuses*.

[3] Keidar Z, Nitecki S (2013). FDG-PET in prosthetic graft infections. *Seminars in Nuclear Medicine*.

[4] Swain TW, Calligaro KD, Dougherty MD (2004). Management of infected aortic prosthetic grafts. *Vascular and Endovascular Surgery*.

[5] Valentine RJ (2001). Diagnosis and management of aortic graft infection. *Seminars in Vascular Surgery*.

[6] Low RN, Wall SD, Jeffrey RB, Sollitto RA, Reilly LM, Tierney LM (1990). Aortoenteric fistula and perigraft infection: evaluation with CT. *Radiology*.

[7] Orton DF, LeVeen RF, Saigh JA (2000). Aortic prosthetic graft infections: radiologic manifestations and implications for management. *Radiographics*.

[8] Kumar R, Basu S, Torigian D, Anand V, Zhuang H, Alavi A (2008). Role of modern imaging techniques for diagnosis of infection in the era of 18F-fluorodeoxyglucose positron emission tomography. *Clinical Microbiology Reviews*.

[9] Mark A, Moss AA, Lusby R, Kaiser JA (1982). CT evaluation of complications of abdominal aortic surgery. *Radiology*.

[10] Fukuchi K, Ishida Y, Higashi M (2005). Detection of aortic graft infection by fluorodeoxyglucose positron emission tomography: comparison with computed tomographic findings. *Journal of Vascular Surgery*.

[11] Macedo TA, Stanson AW, Oderich GS, Johnson CM, Panneton JM, Tie ML (2004). Infected aortic aneurysms: imaging findings. *Radiology*.

[12] Sueyoshi E, Sakamoto I, Kawahara Y, Matsuoka Y, Hayashi K (1998). Infected abdominal aortic aneurysm: early CT findings. *Abdominal Imaging*.

[13] Keidar Z, Engel A, Hoffman A, Israel O, Nitecki S (2007). Prosthetic vascular graft infection: the role of 18F-FDG PET/CT. *Journal of Nuclear Medicine*.

[14] Lauwers P, Van Den Broeck S, Carp L, Hendriks J, Van Schil P, Blockx P (2008). The use of positron emission tomography with (18)f-fluorodeoxyglucose for the diagnosis of vascular graft infection. *Angiology*.

[15] Spacek M, Belohlavek O, Votrubova J, Sebesta P, Stadler P (2009). Diagnostics of “non-acute” vascular prosthesis infection using ^18^F-FDG PET/CT: our experience with 96 prostheses. *European Journal of Nuclear Medicine and Molecular Imaging*.

[16] Bruggink JLM, Glaudemans AWJM, Saleem BR (2010). Accuracy of FDG-PETeCT in the diagnostic work-up of vascular prosthetic graft infection. *European Journal of Vascular and Endovascular Surgery*.

[17] Tokuda Y, Oshima H, Araki Y (2013). Detection of thoracic aortic prosthetic graft infection with 18F-fluorodeoxyglucose positron emission tomography/computed tomography. *European Journal of Cardio-Thoracic Surgery*.

[18] Signore A, Chianelli M, D'Alessandria C, Annovazzi A (2006). Receptor targeting agents for imaging infammatin/infection: where are we now?. *Quarterly Journal of Nuclear Medicine and Molecular Imaging*.

[19] Bakheet SM, Powe J (1998). Benign causes of 18-FDG uptake on whole body imaging. *Seminars in Nuclear Medicine*.

[20] Stumpe KDM, Dazzi H, Schaffner A, Von Schulthess GK (2000). Infection imaging using whole-body FDG-PET. *European Journal of Nuclear Medicine*.

[21] de Winter F, Vogelaers D, Gemmel F, Dierckx R (2002). Promising role of 18-F-fluoro-D-deoxyglucose positron emission tomography in clinical infectious diseases. *European Journal of Clinical Microbiology and Infectious Diseases*.

[22] Bruggink JLM, Slart RHJA, Pol JA, Reijnen MMPJ, Zeebregts CJ (2011). Current role of imaging in diagnosing aortic graft infections. *Seminars in Vascular Surgery*.

[23] Disselhorst JA, Bezrukov I, Kolb A, Parl C, Pichler BJ (2014). Principles of PET/MR imaging. *Journal of Nuclear Medicine*.

[24] Glaudemans AW, Quintero AM, Signore A (2012). PET/MRI in infectious and inflammatory diseases: will it be a useful improvement?. *European Journal of Nuclear Medicine and Molecular Imaging*.

[25] Saleem BR, Berger P, Zeebregts CJ, Slart RH, Verhoeven EL, van den Dungen JJ (2008). Periaortic endograft infection due to *Listeria monocytogenes* treated with graft preservation. *Journal of Vascular Surgery*.

[26] Keidar Z, Nitecki S (2009). FDG-PET for the detection of infected vascular grafts. *Quarterly Journal of Nuclear Medicine and Molecular Imaging*.

[27] Cook GJR, Fogelman I, Maisey MN (1996). Normal physiological and benign pathological variants of 18-fluoro-2-deoxyglucose positron-emission tomography scanning: potential for error in interpretation. *Seminars in Nuclear Medicine*.

[28] Hagerty RD, Salzmann DL, Kleinert LB, Williams SK (2000). Cellular proliferation and macrophage populations associated with implanted expanded polytetrafluoroethylene and polyethyleneterephthalate. *Journal of Biomedical Materials Research*.

[29] Salzmann DL, Kleinert LB, Berman SS, Williams SK (1999). Inflammation and neovascularization associated with clinically used vascular prosthetic materials. *Cardiovascular Pathology*.

[30] van Bilsen PHJ, Popa ER, Brouwer LA (2004). Ongoing foreign body reaction to subcutaneous implanted (heparin) modified Dacron in rats. *Journal of Biomedical Materials Research A*.

[31] Wassélius J, Malmstedt J, Kalin B (2008). High 18F-FDG uptake in synthetic aortic vascular grafts on PET/CT in symptomatic and asymptomatic patients. *Journal of Nuclear Medicine*.

[32] Keidar Z, Pirmisashvili N, Leiderman M, Nitecki S, Israel O (2014). ^18^F-FDG uptake in noninfected prosthetic vascular grafts: incidence, patterns, and changes over time. *Journal of Nuclear Medicine*.

[33] Torizuka T, Zasadny KR, Wahl RL (1999). Diabetes decreases FDG accumulation in primary lung cancer. *Clinical Positron Imaging*.

[34] Gorenberg M, Hallett WA, O'Doherty MJ (2002). Does diabetes affect [^18^F]FDG standardised uptake values in lung cancer?. *European Journal of Nuclear Medicine*.

[35] Diederichs CG, Staib L, Glatting G, Beger HG, Reske SN (1998). FDG PET: elevated plasma glucose reduces both uptake and detection rate of pancreatic malignancies. *Journal of Nuclear Medicine*.

[36] Rabkin Z, Israel O, Keidar Z (2010). Do hyperglycemia and diabetes affect the incidence of false-negative 18F-FDG PET/CT studies in patients evaluated for infection or inflammation and cancer? A comparative analysis. *Journal of Nuclear Medicine*.

[37] Zhuang HM, CortÉs-Blanco A, Pourdehnad M (2001). Do high glucose levels have differential effect on FDG uptake in inflammatory and malignant disorders?. *Nuclear Medicine Communications*.

[38] Kaebnick HW, Bandyk DF, Bergamini TW, Towne JB (1987). The microbiology of explanted vascular prostheses. *Surgery*.

[39] Sharp WJ, Hoballah JJ, Mohan CR (1994). The management of the infected aortic prosthesis: a current decade of experience. *Journal of Vascular Surgery*.

[40] FitzGerald SF, Kelly C, Humphreys H (2005). Diagnosis and treatment of prosthetic aortic graft infections: confusion and inconsistency in the absence of evidence or consensus. *Journal of Antimicrobial Chemotherapy*.

[41] Lankinen P, Lehtimäki K, Hakanen AJ, Roivainen A, Aro HT (2012). A comparative ^18^F-FDG PET/CT imaging of experimental *Staphylococcus aureus* osteomyelitis and *Staphylococcus epidermidis* foreign-body-associated infection in the rabbit tibia. *EJNMMI Research*.

[42] Jamar F, Buscombe J, Chiti A (2013). EANM/SNMMI guideline for ^18^F-FDG use in inflammation and infection. *Journal of Nuclear Medicine*.

[43] Boellaard R, O’Doherty MJ, Weber WA (2010). FDG PET and PET/CT: EANM procedure guidelines for tumour PET imaging: version 1.0. *European Journal of Nuclear Medicine and Molecular Imaging*.

[44] Glaudemans AW, de Vries EF, Galli F, Dierckx RA, Slart RH, Signore A (2013). The use of ^18^F-FDG-PET/CT for diagnosis and treatment monitoring of inflammatory and infectious diseases. *Clinical and Developmental Immunology*.

[45] Miceli MH, Jackson LBJ, Walker RC, Talamo G, Barlogie B, Anaissie EJ (2004). Diagnosis of infection of implantable central venous catheters by [^18^F]fluorodeoxyglucose positron emission tomography. *Nuclear Medicine Communications*.

[46] Singh P, Wiggins B, Sun Y (2010). Imaging of peritoneal catheter tunnel infection using positron-emission tomography. *Advances in Peritoneal Dialysis*.

[47] Bensimhon L, Lavergne T, Hugonnet F (2011). Whole body [^18^F]fluorodeoxyglucose positron emission tomography imaging for the diagnosis of pacemaker or implantable cardioverter defibrillator infection: a preliminary prospective study. *Clinical Microbiology and Infection*.

[48] Turpin S, Lambert R, Poirier N (2010). An unusual looking pacemaker infection imaged with 18F-FDG PET/CT. *European Journal of Nuclear Medicine and Molecular Imaging*.

